# Beyond Communication: Expressing the Language of Humanity in Medicine

**DOI:** 10.5334/pme.2136

**Published:** 2026-02-23

**Authors:** Sandra Apondo

**Affiliations:** 1 Team Lead Curriculum and Faculty Development, TUM Medical Education Center, TUM School of Medicine and Health, Technical University Munich, Germany

## Abstract

Written from the author’s multiple perspectives as a medical educator, physician, and cancer survivor, this reflective piece explores professional identity formation as a process that extends beyond the acquisition of communication skills, drawing attention to the distinction between language and communication. By weaving together educational practice and lived experience, the text invites readers to pause and consider how becoming a doctor is a deeply personal process that requires space for reflection, uncertainty, and the search for one’s own language and meaning, in teaching as well as in our personal and professional lives.

“*Music speaks where words fail*,” said Hans Christian Andersen, whose fairy tales have touched millions, myself included. According to my mother, at age four I could ‘read’ *The Ugly Duckling*, holding the illustrated book, reciting the story of the grey, rejected duckling that became a swan, turning the pages at exactly the right moments. Before I could read letters, I could read musical notes and play the piano. I soon realised Andersen was right.

By 15, I was spending more time practising the piano than attending school, convinced that most of what we learned was useless and that only music could save us. Music is a universal language, translating ineffable feelings into sound. In the sequences of sounds that Beethoven and Bach (among many others) have transcribed for us, all the pain and hope of humanity are condensed. As musicians we pour our own joy, sorrow and longing into their compositions. Music speaks without words, giving voice to what we might otherwise pass over in silence.

Andersen was born in Odense, a city I return to each semester as a visiting scholar at the University of Southern Denmark. I work with colleagues in the humanities to help medical students grow into their professional identity. Should we read Tolstoy with them? Listen to Brahms? Visit a museum or have them paint? Or is it enough to simply sit with them and ask: *Who are you? What does becoming a doctor mean to you?* There are many paths to the subjective, and these paths often take detours through art, literature, or philosophy. Some students prefer more emotional approaches; others lean toward more cognitive ones. What is central in the question we pose to them is the emphasis on *you*: Who are *you*? What does becoming a doctor mean to *you*? We are not seeking a rational analysis of the type they are accustomed to in the natural sciences, in which the objective and rational are front and centre. Rather, we aim to nudge them into unfamiliar, subjective, potentially intimidating terrain. *What does it mean for you personally, at your core?* If we take this question seriously, we must as educators also ask it of ourselves.

*How did I become who I am? And where do I want to grow next?* The answer goes beyond academic achievements or titles. Identity is always both professional and personal. If we genuinely intend to nurture professional identity formation in medical school, we must also be prepared to engage with students’ responses on a deeply personal level. These may include moments of profound doubt, where a student may feel utterly disillusioned with the profession we strive to induct them into – perhaps even to the point where they leave it altogether – or pursue it in ways we know we’re ill-equipped to support.

For my own part, it was doubt that first led me to medicine. Doubt about the omnipotence of music. I gradually sobered up from my youthful intoxication and allowed myself to consider that *perhaps there is something else in the world besides music*. I saw that, above all, there is much suffering in the world and my calling was born: I wanted to become a psychiatrist, to help those who suffer from themselves and the pain of the world, sometimes so strongly they wish to leave it behind. As a doctor, I had sworn an oath to save lives and it seemed to me that the most meaningful task of all was to help people want to go on living *despite* everything, perhaps even to be able to live well. Over a decade of clinical practice, I succeeded in helping many with medication, through psychotherapy, and, above all, by accompanying them as they rediscovered or found anew something within themselves that made life worth living. For many, it was music, literature, art, spirituality and, most profoundly, connection with others and meaningful work.

Ultimately, it comes down to expressing oneself in the world, and that expression need not be verbal. It can be musical, spiritual, or take any artistic form. After all, we are all the artists of our own lives if we are willing to live them. As long as a form of expression remains, there is life in a human being. When suffering is met with silence, life begins to wither, especially in medicine, where isolation and unspoken pain are all too common. We know this from our own profession, which year on year ranks high in the suicide statistics. Most physicians can probably tell stories of colleagues who were suddenly no longer there, and of whom one ‘never would have thought’ that they would drown their cheerfulness and loyalty, and life-saving expertise in depression and addictions. The medical profession is, therefore, a dangerous one. The reasons for this are well known.

As medical educators, we aim to equip future doctors with the skills and knowledge to deliver safe patient care, so much so that we can trust them with our own lives. But our ambition exceeds this: we also want them to be empathetic, ethical, collaborative and responsible professionals. In short, we hope they become good people. Yet the weight of this ideal can crush. One can break under it.

Professional identity formation must therefore also actively support young medical students in coming to understand themselves more deeply. Discovering who we are may also be revealed in how we express ourselves through our principal clinical activities. For the vascular surgeon, through their deft fingers and precise eye. For the psychiatrist, through a keen listening ear and deliberately chosen words. For the oncologist, through chemotherapy and the hope that it will bring healing or at least reduce suffering. Whatever the form, all these expressions of healing attain meaning only in the mirror of the patient. Without the suffering human as the counterpart, the medical part of the professional cannot express itself. But since the physician’s professional identity is most closely intertwined with their personal identity (or rather, identities), the human, the deeply personal, always expresses itself in the medical as well. To the misfortune of patients, however, the human aspect in the medical often recedes into the background. We see the white coat and, as our lives may depend on it, we trust in the skill it represents. But in this act of trust, we often lose sight of the person wearing it, of who they truly are. In that moment, connection fades, not just between people, but within ourselves.

As a cancer patient, I was the counterpart to many medical professionals. I can affirm that most physicians are friendly communicators and apply the rules they were taught in communication training. At the first encounter they introduce themselves with their own name and look me in the eyes, they begin the ward round with an open question and make a visible effort not to interrupt me after 20 seconds. They sit down at a table with me for the informed consent conversation and kindly but firmly deflect the disturbing phone call. On the surface, they do everything correctly – a perfect score in the OSCE. But this is no exam, no simulation. This is real life between two people, neither of whom is role-playing or pretending. We are real human beings, and for each other in that moment the only counterpart that matters.

Objectively speaking, the doctor chose his words well, provided all the necessary information and was polite. But as a patient, I felt something entirely different. Only after the conversation ended did I notice a lingering emptiness and loneliness. For a long time, I thought it was my fault. Perhaps I had expected too much or failed to express what I truly needed. I tried to appear strong, even though I felt small and vulnerable. Despite being technically well-executed, the conversation didn’t feel like a genuine human exchange.

Through music, reading, and writing, my emptiness began to fade. I found a way to express what illness truly means to me. This personal language reconnects me with myself, reminding me that I’m more than a patient; I’m a whole human being. Without that connection to my own humanity, I can’t truly connect with my medical counterpart. We may speak politely but have nothing real to say. I believe doctors feel this too, that efficient communication doesn’t always make for a meaningful conversation. They may leave the room with the same stale emptiness. Over time, these moments accumulate. Sometimes that emptiness surfaces as depression or is numbed with alcohol. We hope it also finds expression, whether through the steady rhythm of chopping wood for winter, the splendour of the opera, the strain of lifting weights, or the quiet of a book. These acts help us return to ourselves and the people who shape our professional identity. In those moments – a silence, a smile, a resonance – we glimpse the personal side of professionalism, and the emptiness begins to fill.

Perhaps then we have said more to each other than we have actually communicated.

